# Comparative Analysis of Micro-Computed Tomography and 3D Micro-Ultrasound for Measurement of the Mouse Aorta

**DOI:** 10.3390/jimaging10060145

**Published:** 2024-06-17

**Authors:** Hajar A. Alenezi, Karen E. Hemmings, Parkavi Kandavelu, Joanna Koch-Paszkowski, Marc A. Bailey

**Affiliations:** Leeds Institute for Cardiovascular and Metabolic Medicine, School of Medicine, University of Leeds, Leeds LS2 9JT, UKm.a.bailey@leeds.ac.uk (M.A.B.)

**Keywords:** Micro-CT, ultrasound, preclinical imaging, aortic aneurysm

## Abstract

Aortic aneurysms, life-threatening and often undetected until they cause sudden death, occur when the aorta dilates beyond 1.5 times its normal size. This study used ultrasound scans and micro-computed tomography to monitor and measure aortic volume in preclinical settings, comparing it to the well-established measurement using ultrasound scans. The reproducibility of measurements was also examined for intra- and inter-observer variability, with both modalities used on 8-week-old C57BL6 mice. For inter-observer variability, the μCT (micro-computed tomography) measurements for the thoracic, abdominal, and whole aorta between observers were highly consistent, showing a strong positive correlation (R^2^ = 0.80, 0.80, 0.95, respectively) and no significant variability (*p*-value: 0.03, 0.03, 0.004, respectively). The intra-observer variability for thoracic, abdominal, and whole aorta scans demonstrated a significant positive correlation (R^2^ = 0.99, 0.96, 0.87, respectively) and low variability (*p*-values = 0.0004, 0.002, 0.01, respectively). The comparison between μCT and USS (ultrasound) in the suprarenal and infrarenal aorta showed no significant difference (*p*-value = 0.20 and 0.21, respectively). μCT provided significantly higher aortic volume measurements compared to USS. The reproducibility of USS and μCT measurements was consistent, showing minimal variance among observers. These findings suggest that μCT is a reliable alternative for comprehensive aortic phenotyping, consistent with clinical findings in human data.

## 1. Introduction

Aortic aneurysm is a term used to describe an enlargement of the aorta greater than 1.5 times its normal size. The causes and prevalence of aneurysms vary among different sections of the aorta [1,2,3,4] with significant structural modifications such as elevated collagen, reduced elastin, and loss of smooth muscle cells associated with the formation of aortic aneurysms. Aneurysms tend to grow in size over time with an eventual risk of rupture posing a serious threat to life, limb perfusion, and the functioning of vital organs [2,5,6]. Compared to thoracic aortic aneurysms or dissections, abdominal aortic aneurysms (AAA) are three times more common [7,8]. Most AAAs develop in the infrarenal abdominal aorta as a degenerative disease that is commonly associated with older age, hypertension, smoking, and genetic predisposition [9,10,11]. In the clinical setting, both ultrasound (USS) and CT angiography (CT) are commonly used to monitor AAA and plan treatment, with CT-derived measures generally larger than those from USS [12,13,14,15]. Surgical repair is recommended once the AAA reaches 5.5 cm in diameter on an USS.

Preclinical AAA models are useful for understanding disease pathology and assessing new mechanisms and potential novel treatments. In the preclinical setting, µUSS can be used to derive three-dimensional lumen volume (3DLV) measurements that are more sensitive to changes in aneurysm size compared to two-dimensional (2D) measurements of maximal diameter [16].

Micro-computed tomographic angiography (μCTA) is another valuable tool available in the preclinical setting [17]. μCTA can generate three-dimensional (3D) tomographic data at a microscopic level of resolution (with a voxel size of 100 mm^3^) by capturing a multitude of two-dimensional (2D) projection series from different angles surrounding the subject [17,18]. This study assessed the intra- and inter-observer variability of μCTA-generated 3D aortic volume reconstructions and compared them to the 3DLV obtained using µUSS. Our investigation compared the assessment procedures for suprarenal and infrarenal aortic segments using μCT and USS, revealing methodological variations influenced the results. The main difference between these methods is the positioning of the animals during imaging. Specifically, for USS imaging, the mice were placed in the supine position and stretched out, whereas for μCT scanning, they were positioned in the prone position. It is important to emphasise the importance of comparing different imaging techniques comprehensively to fully comprehend their specific advantages and drawbacks when evaluating aortic measurements.

## 2. Materials and Methods

### 2.1. Mice

Male C57BL6/J mice, obtained from Jackson laboratory stock #:000664, were used for experiments at 8 weeks of age. Following the UK Animals Scientific Procedures Act 1986, experiments were conducted under Home Office project license PP8169223 (4 August 2021). Ethical approval was granted by the Animal Research and Ethics Board at the University of Leeds. The mice were housed in ventilated cages (GM500, Techniplast, West Chester, PA, USA, five mice per cage) with a 12 h light/dark cycle, 50–70% humidity, and a temperature of 21 °C. They received a standard chow diet (CRM, Special Diet Services, Augy, France) and had access to water through Hydropac^®^, London, UK, pouches. Any animal showing signs of distress or suffering was humanely euthanised.

### 2.2. Micro-Ultrasound Scan (µUSS)

3DLV measures of the abdominal aorta were obtained as per Waduud and colleagues [16]. Briefly, mice were anaesthetised using isoflurane, positioned supine on the USS table, and imaged on a Vevo 2100 µUSS system (VisualSonics, Bothell, WA, USA) with a 40 MHz frequency MS-550D transducer. A 12 mm ROI above the left renal artery was measured towards the diaphragm to measure the suprarenal aorta and 12 mm down from the left renal artery towards the aortic bifurcation to measure the infrarenal aorta. Images were captured with respiration gating at the peak systole for 157 frames and reconstructed using VevoLab software version 1.7.0 for 3DLV derivation. The evaluation of inter-observer variability in USS was excluded because it has been extensively examined in a recent publication by Waduud et al. [16], who assessed both intra- and inter-observer variability in identifying the aorta via USS. The inclusion of this analysis in our study would have been redundant. Therefore, to avoid repetition, we excluded it from this paper.

### 2.3. Micro-Computed Tomographic Angiography (μCTA)

The mice were anaesthetised with isoflurane and 100 µL of Exitron Nano 12,000 contrast agent (Miltenyi Biotech, Bergisch Gladbach, Germany) was administered via the lateral tail vein. The mouse was positioned supine for imaging by μCTA on a Skyscan 1176 (Bruker, San Jose, CA, USA) pre-clinical CT system. This device has an 11-megapixel cooled digital X-ray camera that connects through fibre optics to a scintillator X-ray detector. A 35.98 µm pixel size, an AL 0.5 mm filter, 50 kV, and 476 µA scanning parameters were used during the scan. This device can non-invasively reconstruct any cross-section of the animal’s anatomy, measure internal structural characteristics, and create a three-dimensional image from the reconstructed data.

Nrecon v.1.7.4 and CT analyzer (CTan) software v.1.18 (Bruker, USA) were used for the post-processing reconstruction. The reconstruction parameters used included a smoothing of 4, a ring artefact reduction of 14, and a beam hardening of 40%.

The edge of the mouse was identified by mouse volume of interest and anisotropic diffusion image processing was carried out in CTan to remove noise yet maintain the sharpness of the image. Regions of interest (ROIs) were manually highlighted every 5 slices from the start of the ascending aorta to the end of the abdominal aorta, covering the bifurcation. To determine the aortic volume measurements for the thoracic, abdominal, and whole aorta, a three-dimensional (3D) analysis was performed. For the analysis, the aortic root to the diaphragm is referred to as the thoracic aorta. The diaphragm to aortic bifurcation is referred to as the abdominal aorta. The combined measures from the ascending aorta to aortic bifurcation are termed whole aorta. The μCT anterior–posterior maximal diameters (APmax) of the suprarenal aorta and the infrarenal aorta were measured using CTan software. To measure the APmax of the suprarenal aorta, we located the left renal artery and then measured −6 mm from it. We selected the slice with the largest diameter and measured the APmax.

For the infrarenal artery, the APmax was measured by selecting the slice with the largest aortic diameter just below the left renal artery.

To measure the APmax by USS, we visually identified the largest diameter aorta in the supra- and infrarenal regions using the Vevolab 2100 software.

### 2.4. Statistical Analysis

The mean and standard deviation were used to summarize the data, and scatter plots with lines of best fit were used to assess disparities. The medians and interquartile ranges (IQR) were measured to provide a more accurate description of the data distribution.

A 95% confidence level and a *p*-value of 0.05 are considered statistically significant. GraphPad Prism version 8.0 was used for statistical analysis. Bland–Altman plots were used to assess the agreement between two measurements, specifically by detecting bias and limits of agreement. It is used to evaluate both intra- and inter-observer variability in measurements obtained from μCT and USS. The reproducibility of observer measurements was evaluated using Bland–Altman plots and limits of agreement. The Pearson’s correlation is employed to evaluate the linear association between two variables. It is used to assess the consistency of intra- and inter-observer measurements, as well as to compare measurements obtained from μCT and USS. The Mann–Whitney U test was used to compare the medians of two independent groups. It is especially well-suited for small sample sizes. Two-sample *t*-tests were used to assess the means of two distinct groups, such as μCT vs. USS measurements, and identify any statistically significant disparities between their means. Linear regression analysis is used to model the relationship between two variables, specifically to assess the correlation between μCT and USS readings and determine the level of connection.

## 3. Results

### 3.1. Inter-Observer and Intra-Observer Variability in μCT

**Figure 1:**
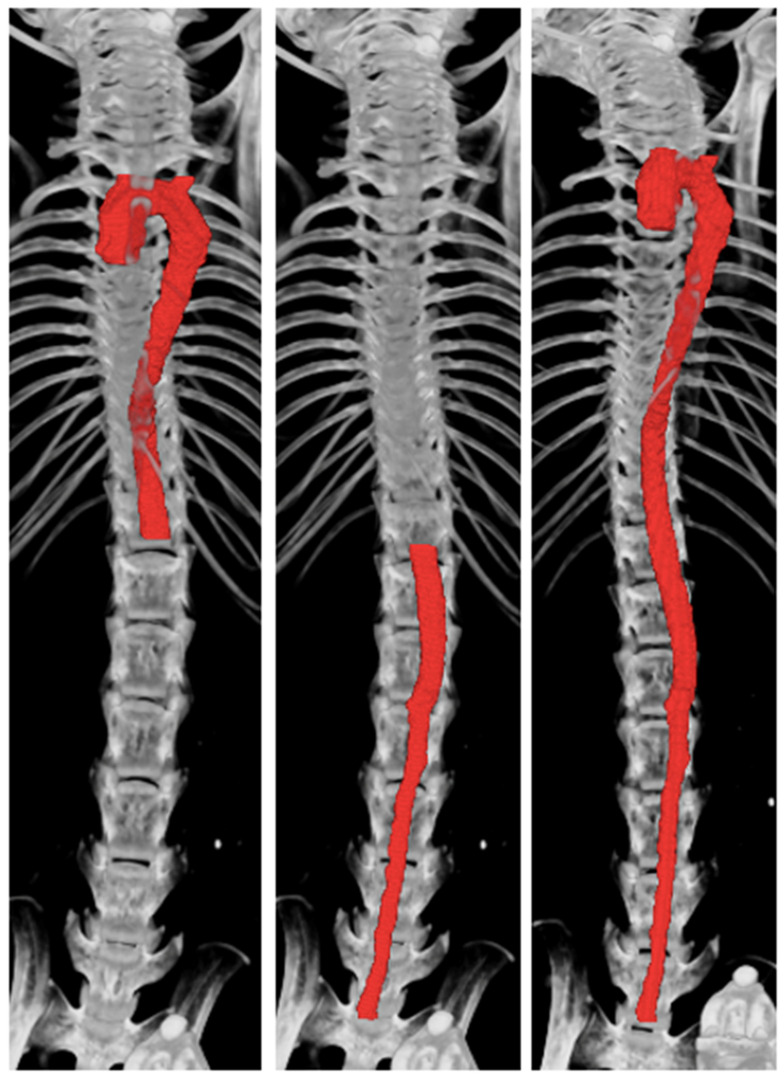
Micro-computed tomography (μCT) reconstruction of the thoracic, abdominal, and whole aorta using CTan.

Two independent observers generated the 3D volumes of the aorta from the raw μCT images from five mice. Examples of μCT image reconstruction are shown in Figure 1.

#### 3.1.1. Inter-Observer Variability

In this study, we evaluated inter-observer variability in 3D aortic volume measurements using μCT by comparing data from two independent observers.

**Figure 2:**
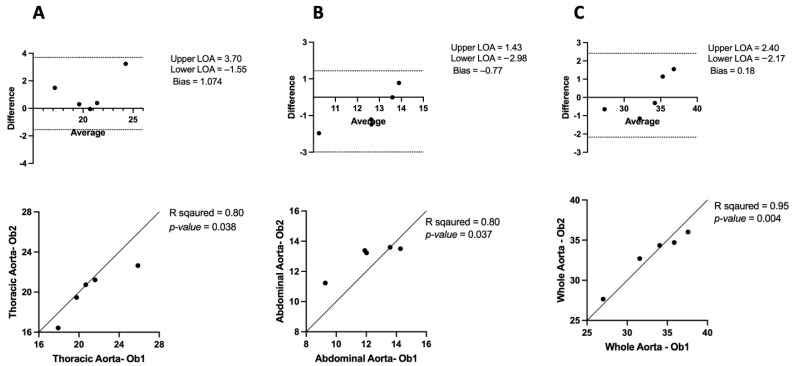
Bland–Altman plots and correlation analysis of inter-observer variability in μCT: in (**A**) thoracic (**B**) Abdominal and (**C**) whole aorta.

The thoracic aorta measurements showed a strong positive correlation (R^2^ = 0.80, *p* = 0.03) between observers but no significant inter-observer variability (bias: 1.074 mm, Limits of agreement (LOA): 3.70 to −1.55) (Figure 2A). Similarly, the abdominal aorta measurement demonstrated a positive correlation (R^2^ = 0.80, *p* = 0.03) and no significant difference in the inter-observer variability (bias: −0.77 mm, LOA: 1.43 to −2.98) (Figure 2B). For the whole aorta, there was a strong positive correlation (R^2^ = 0.95, *p* = 0.004) and no significant difference in the inter-observer variability (bias: 0.11 mm, LOA: 2.40 to −2.17) (Figure 2C). These findings indicate a significant level of agreement between observers, highlighting the reproducibility and reliability of μCT for aortic measurements.

The Mann–Whitney U test, which is suitable for small sample sizes, was used to compare inter-observer variability between Observer 1 and Observer 2 across different sections of the aorta.

For the thoracic aorta, the median for Observer 1 was 20.68 (IQR: 18.85–23.74), and for Observer 2, it was 20.73 (IQR: 17.95–21.93). The test showed no significant difference between the observers (U = 11, *p* = 0.84).

In the abdominal aorta, the median for Observer 1 was 12.02 (IQR: 10.59–13.94), and for Observer 2, it was 13.38 (IQR: 12.23–13.55). The test indicated no significant difference between the observers (U = 11, *p* = 0.84).

For the whole aorta, the median for Observer 1 was 30.04 (IQR: 29.28–36.71), and for Observer 2, it was 34.34 (IQR: 30.18–35.37). The test again showed no significant difference between the observers (U = 12, *p* > 0.99).

#### 3.1.2. Intra-Observer Variability

**Figure 3:**
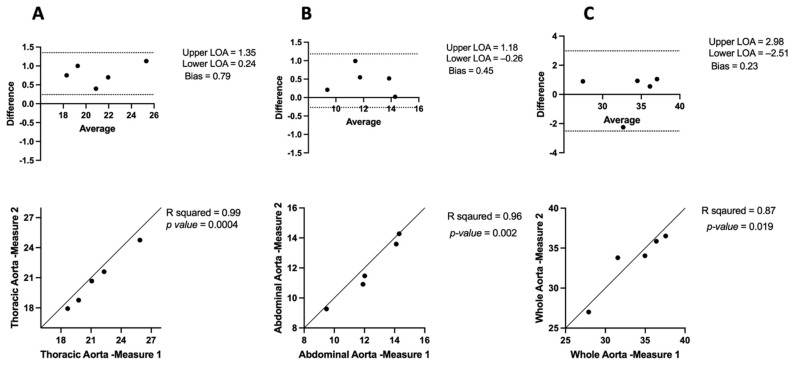
Bland–Altman plots and correlation analysis of intra-observer variability in μCT: (**A**) in the thoracic aorta, (**B**) abdominal aorta, and (**C**) whole aorta.

To assess intra-observer variability, the same observer evaluated intra-observer variability on two consecutive occasions by reconstructing the μCT 3D measurements from the raw data (Figure 3). The results showed a strong positive correlation for the thoracic aorta (R^2^ = 0.99, *p* = 0.0004) with no noticeable intra-observer variability (bias: 0.79 mm, LOA: 1.35 to 0.24) (Figure 3A). For the abdominal aorta, the correlation was also strong (R^2^ = 0.96, *p* = 0.002) with minimal intra-observer variability (bias: 0.45 mm, LOA: 1.18 to −0.26) (Figure 3B). The whole aorta measurement exhibited a strong positive correlation (R^2^ = 0.87, *p* = 0.01) with no significant intra-observer variability (bias: 0.23 mm, LOA: 2.98 to −2.51) (Figure 3C). These results indicate high consistency and reproducibility of μCT measurements when conducted by the same observer.

The Mann–Whitney U test was used for intra-observer variability between Measurement 1 and Measurement 2 across different sections of the aorta.

For the thoracic aorta, the median for Measurement 1 was 21.08 (IQR: 19.22–24.09), and for Measurement 2, it was 20.68 (IQR: 18.35–23.18). The test showed no significant difference between the observers (U = 10, *p* = 0.69).

In the abdominal aorta, the median for Measurement 1 was 12.02 (IQR: 10.69–14.21), and for Measurement 2, it was 11.47 (IQR: 10.09–13.94). The test indicated no significant difference between the observers (U = 9, *p* = 0.55).

For the whole aorta, the median for Measurement 1 was 34.97 (IQR: 29.73–36.99), and for Measurement 2, it was 34.04 (IQR: 30.41–36.19). The test again showed no significant difference between the observers (U = 11, *p* = 0.84).

### 3.2. Comparing μCT and USS Aortic Measures

**Figure 4:**
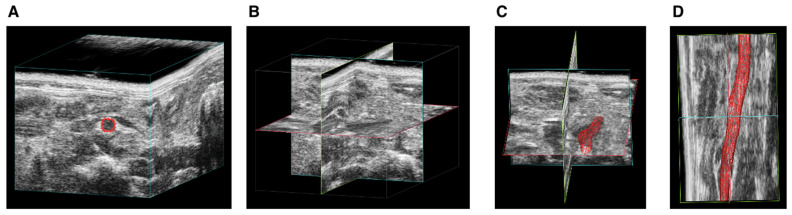
Steps of ultrasound (USS) three-dimensional lumen volume measurement (3DLV) reconstruction of the suprarenal and infrarenal aorta. (**A**) Cube view of the infrarenal aorta, (**B**) cross view, (**C**) surface view, and (**D**) overlay view.

**Figure 5:**
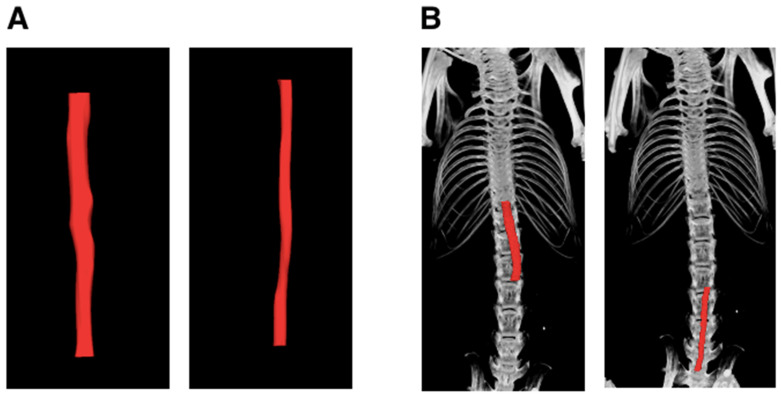
Images of suprarenal and infrarenal aortas reconstruction in (**A**) ultrasound (USS); suprarenal (left) and infrarenal (right) and (**B**) micro-computed tomography (μCT); suprarenal (left) and infrarenal (right).

**Figure 6:**
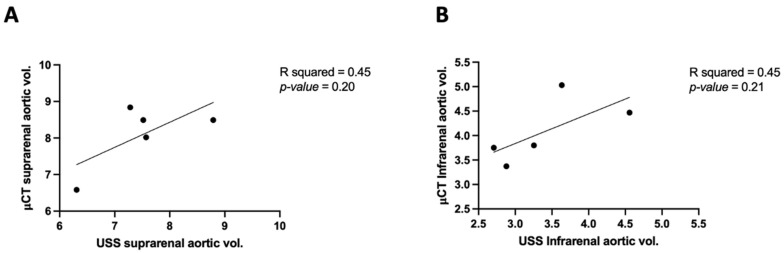
Correlation of Micro-CT (μCT) 3D volume against ultrasound scan (USS): the aortic volume measurements of μCT and USS in (**A**) the suprarenal aorta (**B**) and the infrarenal aorta.

Aortic volume comparisons between μCT and USS in mice indicated that μCT tends to yield larger measurements than USS for both suprarenal and infrarenal segments. Example images of USS reconstruction in mice are shown in Figure 4. The mean differences were 0.59 mm^3^ (suprarenal) and 0.67 mm^3^ (infrarenal), but these were not statistically significant, with *p*-values of 0.32 and 0.16, respectively. Moderate positive correlations were observed in both segments (R^2^ = 0.45), suggesting a consistent but not strong relationship between the μCT and USS measurement techniques (Figure 5 and Figure 6). The measurements of aortic volume in the supra- and infrarenal aorta, obtained using both USS and μCT, are reported in Table 1.

### 3.3. APmax Inter-Observer and Intra-Observer Variability in μCT

#### 3.3.1. APmax Intra-Observer Variability in μCT

**Figure 7:**
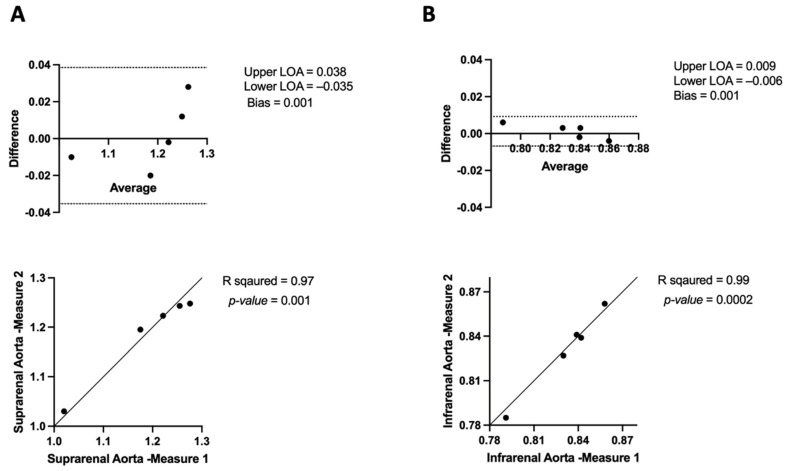
Bland–Altman plots and correlation assessment of μCT intra-observer variability for anterior–posterior maximum aortic diameter measurements (APmax) in (**A**) suprarenal aorta and (**B**) in the infrarenal aorta.

The intra-observer variability of the anterior–posterior maximum diameter (APmax) of the suprarenal aorta was measured using Bland–Altman analysis. The results showed (bias: 0.001 mm, LOA: −0.035 to 0.038) (Figure 7A). These narrow ranges of agreement demonstrate a high degree of agreement between repeated measurements taken by the same observer and minimal variability. Additionally, a statistically significant and strong positive correlation (R^2^ = 0.97, *p* = 0.001) was found by Pearson’s correlation analysis between the first and second measures of APmax in the suprarenal aorta (Figure 7A).

When measuring the intra-observer variability of the maximum diameter (APmax) of the infrarenal aorta, the Bland–Altman plot revealed a mean bias of 0.001, with limits of agreement ranging from −0.006 to 0.009 (Figure 7B). These findings demonstrate that the two measurement sets have a high degree of agreement. Additionally, a high positive association that was statistically significant with an almost perfect correlation (R^2^ = 0.99, *p* = 0.0002) was revealed by Pearson’s correlation analysis between repeated measurements (Figure 7B).

The Mann–Whitney U test was used to assess APmax intra-observer variability between Measurement 1 and Measurement 2 across the suprarenal and infrarenal aorta. For the suprarenal aorta, the median for Measurement 1 was 1.22 (IQR: 1.09–1.26), and for Measurement 2, it was 1.22 (IQR: 1.11–1.24). The test showed no significant difference between the observers (U = 12, *p* > 0.99). In the infrarenal aorta, the median for Measurement 1 was 0.83 (IQR: 0.81–0.85), and for Measurement 2, it was 0.83 (IQR: 0.80–0.85). The test indicated no significant difference between the observers (U = 11.5, *p* = 0.88).

#### 3.3.2. APmax Inter-Observer Variability in μCT

**Figure 8:**
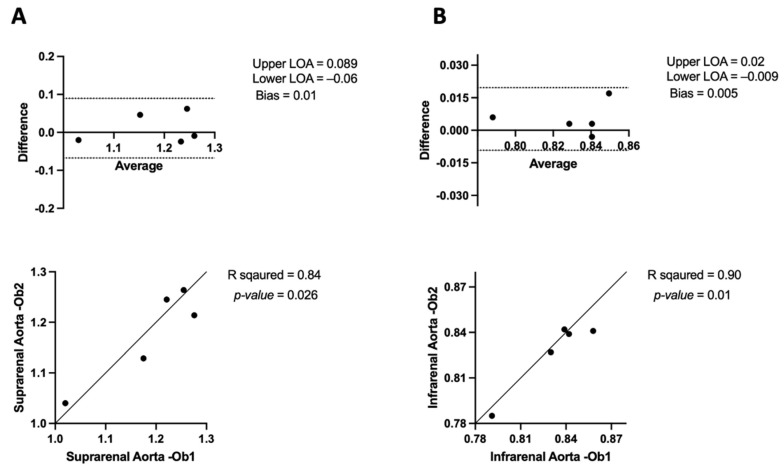
Bland–Altman plots and correlation assessment of μCT inter-observer variability for anterior–posterior maximum aortic diameter measurements (APmax) in (**A**) suprarenal aorta and (**B**) in infrarenal aorta.

The Bland–Altman analysis for the anterior–posterior maximum diameter (APmax) of the suprarenal aorta indicates consistent inter-observer readings, with a modest mean bias of 0.01 and limits of agreement ranging from −0.06 to 0.089. Additionally, Pearson’s correlation analysis demonstrates a strong positive association with statistical significance (R^2^ = 0.84, *p* = 0.026), indicating the validity of repeated measurements (Figure 8A). The Bland–Altman plot for the infrarenal aorta shows a low mean bias of 0.005 and narrow margins of agreement ranging from −0.009 to 0.02, suggesting high reliability for intra-observer measurements. Pearson’s correlation analysis supports this, with a near–perfect correlation with statistical significance (R^2^ = 0.90, *p* = 0.01), indicating great reliability for the APmax of the infrarenal aorta (Figure 8B).

The Mann–Whitney U test was used to assess APmax inter-observer variability between Observer 1 and Observer 2 across the suprarenal and infrarenal aorta. For the suprarenal aorta, the median for Observer 1 was 1.22 (IQR: 1.09–1.26), and for Observer 2, it was 1.21 (IQR: 1.08–1.25). The test showed no significant difference between the observers (U = 11, *p* = 0.84). In the infrarenal aorta, the median for Observer 1 was 0.83 (IQR: 0.81–0.85), and for Observer 2, it was 0.83 (IQR: 0.80–0.84). The test indicated no significant difference between the observers (U = 10, *p* = 0.65).

### 3.4. APmax in USS Intra-Observer Variability

**Figure 9:**
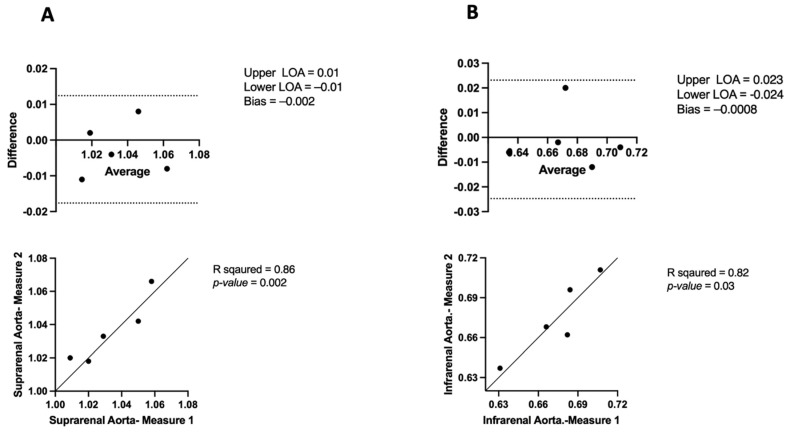
Bland–Altman plots and correlation evaluation of USS intra-observer variability in measurements of the anterior–posterior maximum aortic diameter in (**A**) suprarenal aorta and (**B**) infrarenal aorta.

The Bland–Altman plot for intra-observer variability in APmax of suprarenal aortic diameter measurements showed high consistency with LoAs ranging from −0.017 to 0.01, and a minor mean bias (−0.002). This was supported by a significant Pearson correlation (R2 = 0.86, *p* = 0.02) (Figure 9A). The Bland–Altman plot of APmax for the infrarenal aorta shows limited agreement (−0.024–0.023) and a small mean bias (−0.0008). The Pearson correlation graph showed a strong intraobserver correlation (R^2^ = 0.827, *p* = 0.03) (Figure 9B). Additionally, the comparison of APmax measurements between the suprarenal and infrarenal aortic regions using different modalities demonstrated significant correlations and reliability of these measurements (Table 2).

The Mann–Whitney U test was used to assess APmax intra-observer variability between Measurement 1 and Measurement 2 across the suprarenal and infrarenal aorta. For the suprarenal aorta, the median for Measurement 1 was 1.02 (IQR: 1.01–1.05), and for Measurement 2, it was 1.03 (IQR: 1.01–1.05). The test showed no significant difference between the observers (U = 11.50, *p* = 0.88). In the infrarenal aorta, the median for Measurement 1 was 0.68 (IQR: 0.64–0.69), and for Measurement 2, it was 0.66 (IQR: 0.64–0.70). The test indicated no significant difference between the observers (U = 12, *p* > 0.99).

### 3.5. APmax in μCT vs. USS

**Figure 10:**
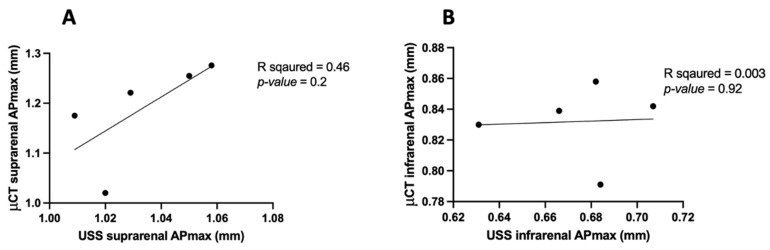
A comparison of anterior–posterior maximal diameters (APmax) was made using micro-computed tomography (μCT) and ultrasound (USS) in the (**A**) suprarenal aorta and (**B**) infrarenal aorta.

In the comparison of the anterior–posterior maximum diameter (APmax) measurements of aorta volume obtained by μCT and USS, we observed distinct differences between the suprarenal and infrarenal aortic regions. In the suprarenal aorta, there was a positive linear correlation between μCT and USS volumes (R^2^ = 0.46, *p* = 0.2) (Figure 10A). However, the infrarenal aortic volume measurements showed no correlation (R^2^ = 0.003, *p* = 0.92) (Figure 10B). The mean differences between μCT and USS APmax were 0.15 mm^3^ (suprarenal) and 0.15 mm^3^ (infrarenal), but these were not statistically significant, with *p*-values of 0.01 and <0.0001, respectively.

## 4. Discussion

This study is the first to offer a detailed comparison of μCT and μUSS for measuring aortic dimensions in mice. While several authors have used these techniques, the comparison of reproducibility and variability between imaging modalities in preclinical settings has not been investigated thoroughly. The study also compares the range of intra-observer and inter-observer variability for μCT and μUSS measurements and provides information about the reliability of these techniques. Thus, this level of detailed comparison for variability and reproducibility between these modalities is a novel contribution.

Our study demonstrates the strengths and limitations of two distinct imaging modalities, providing a theoretical basis for more accurate and dependable measurement techniques in preclinical models of aortic aneurysms. Better assessment of disease development and treatment options would be possible. State-of-the-art imaging technologies including μCTA are used in this study to produce 3D tomographic data with microscopic resolution. This high level of detail improves the accuracy of aortic measurements and leads to a more complete understanding of aortic morphology.

### 4.1. Inter- and Intra-Observer Variability

The use of preclinical micro-computed tomography (μCT) imaging with a contrast agent was employed to precisely measure the total aortic volume in mice. Our results demonstrate that the μCT technique can accurately quantify aortic volume with both high inter-observer and intra-observer agreement. As illustrated in Figure 2, the strong agreement between the measurements taken by the two observers was indicative of low inter-observer variability and high Pearson correlation values (greater than 0.8), indicating a strong linear relationship between the measurements. Additionally, Figure 3 shows significant positive correlations between repeated measurements of the thoracic and abdominal aortic regions, as well as for the whole aorta, with relatively low intra-observer variability. Our findings of intra- and inter-observer variability are consistent with those reported in [16], confirming the reliability of μCT as an imaging method for measuring aortic dimensions.

### 4.2. Comparison of μCT and USS

A positive correlation is shown between the suprarenal aorta volume measurements by µCT and USS in Figure 6. On average the μCT suggests the aorta is 0.59 mm^3^ larger than the USS measurement (*p*-value = 0.32) in the suprarenal aorta. Additionally, the μCT suggests the aorta is 0.67 mm^3^ larger than the USS measure (*p*-value = 0.16) in the infrarenal aorta, as shown in Figure 6. The µCT aortic volume measurement rises in conjunction with the USS aortic volume measurement. This implies that when it comes to assessing aorta volume in the supra- and infrarenal regions, the two imaging modalities are comparatively consistent with one another. Our investigation demonstrated high repeatability, even if the USS is user dependent. We observed a trend towards µCT overestimating aortic volume measures when compared to USS. These findings are in line with other research that looked at similar situations in human clinical practice [15,19,20,21,22,23,24,25]. While μCT and USS-derived aortic volumes differed significantly in absolute terms, our results also aligned with those of a prior study [26] that examined μCT and USS (3D and M-mode USS) AAA in mouse models, as well as that seen in human patients with AAA, where it is well known that CT overestimates aortic size in comparison to USS. Despite the differences in volume measurements across all modalities, there is a correlation [26]. The measuring techniques for the suprarenal and infrarenal aortic segments were different in our comparison between μCT and USS. Inconsistencies between the measurements from the two modalities were caused by these methodological variations in positioning. Moreover, these differences were caused by the mice’s orientation; for USS imaging, the mice were stretched and put supine, whereas for μCT scans, they were placed prone. The USS measurement is standardised at a distance of 12 mm; however, the same measurement cannot be applied to μCT as a 12 mm measurement resulted in excessively high results. This is likely due to the fact that the mice were not stretched flat during the μCT procedure. Volumetric measurements involve calculating the 3D volume of the aorta. The anterior–posterior maximum diameter (APmax) refers to the greatest linear distance between the anterior and posterior walls of the aorta. This measurement is usually obtained at the location where the diameter is the largest. The combination of volumetric and APmax measurements provides a more comprehensive evaluation of the aorta. Volumetric measurements give a detailed assessment of the overall shape and size of the aorta, while APmax is crucial for assessing clinical risk.

When measuring the anterior–posterior maximal diameter (APmax) of the aorta using μCT and USS, we observed minimal variation between observers and within repeated measurements taken by the same observer, as shown in Figure 7, Figure 8, Figure 9 and Figure 10. On average, the μCT APmax suggests the aorta is 0.15 mm^3^ larger than the USS APmax measurement (*p*-value = 0.01) in the suprarenal aorta. Additionally, the μCT APmax suggests the aorta is 0.15 mm^3^ larger than the USS APmax measurement (*p*-value <0.0001) in the infrarenal aorta, as shown in Figure 10. Specifically, Figure 10A shows a moderate positive correlation (R^2^ = 0.46) between the two modalities in the suprarenal aorta. However, this correlation is not statistically significant (*p* = 0.2), indicating that while there is some agreement, it is insufficient to draw definitive conclusions. In contrast, Figure 10B reveals no correlation (R^2^ = 0.003) in the infrarenal aorta, with a *p*-value of 0.92. Several factors may contribute to this lack of correlation. The infrarenal aorta is a smaller and more challenging region to image accurately. Bowel contents, such as faeces, can obscure USS measurements, affecting accuracy and leading to inconsistencies. Unlike USS, μCT remains unaffected by bowel contents, providing a clear advantage.

The differences in imaging techniques and the fundamental characteristics of each modality also play a significant role. μCT can produce more detailed images of the entire aorta, including regions difficult to visualise with ultrasound. This difference in resolution and measurement methods between the two modalities can add to measurement discrepancies. The limitations of USS’s low greyscale resolution, which have been mitigated in more recent systems, also contribute to these differences. Additionally, the positioning of mice during imaging (supine for USS and prone for μCT) may further contribute to measurement discrepancies. These factors suggest potential inconsistencies between the two modalities in this specific segment, indicating a need for further research. The observed variations underscore the importance of careful interpretation and validation of imaging data, particularly in preclinical research, where accurate measurement of aortic dimensions is crucial for studying disease progression and evaluating potential treatments. According to our results, USS and μCT estimates of APmax may differ based on the specific aortic segment being examined and observer influence. The substantial positive correlations and statistically significant results from the Pearson correlation analysis demonstrate the reproducibility of the measures. While μCT provides considerably larger measurements compared to USS, both modalities appear suitable for accurate assessment of aortic dimensions, which is essential for cardiovascular disease research. Due to the high degree of agreement among observers and methodologies, these approaches are well-suited for multi-observer and longitudinal research. Previous studies have demonstrated a high correlation between the USS three-dimensional lumen volume (3DLV) measurement of the murine aorta and more accurate modalities like CT and MRI, as well as histological examination of aortic aneurysms [16,27,28]. This is consistent with our findings, which showed a correlation between USS and μCT for the aortic volume measurements. With μCT, the thoracic aorta can be imaged, volume calculation performed, and architecture evaluated throughout the ascending, arch, and descending thoracic aorta—a task not achievable with USS imaging. Preclinical imaging offers the advantage of allowing for non-invasive animal observation during experimental interventions [29], which bears significant similarities to the clinical setting.

### 4.3. Limitations and Future Directions

The study is limited by its small sample size, which reduces the ability to apply the findings to a larger population and limits the statistical power of the results. Additionally, the use of only 8-week-old C57BL6 mice may not fully capture the variability that exists across different strains or age groups and may not be directly applicable to humans. Measurement variability may also be introduced due to differences in positioning and imaging settings between micro-CT and USS. Moreover, inherent differences in resolution and measurement methods between these two imaging techniques could potentially impact the results. Furthermore, it is important to note that imaging was only conducted at a single time point, which limits our understanding of the reliability of the measurements over time. Finally, the absence of histological examination to confirm the imaging results is another limitation of this study. Further research might enhance imaging techniques, confirm findings by histological examination, and link μCT-derived parameters to clinical outcomes in AAA patients. Developing automated image analysis tools can enhance data processing and incorporate μCT imaging into preclinical and clinical research procedures.

Also, to enhance the statistical power and generalisability of the findings, it is recommended to conduct studies with larger sample sizes. Additionally, performing longitudinal studies would be advantageous to track the progression of aortic changes over time and evaluate the reliability of each imaging technique in dynamic settings.

## 5. Conclusions

μCT is a reproducible method for measuring aortic volume in mice which correlates with USS. There is a trend towards overestimation of aortic volume with μCT compared to USS. A major strength of μCT is the ability to reproducibly measure aortic volume in the root, arch, and descending thoracic abdominal aortas.

## Figures and Tables

**Table 1:** Aortic volume measurements across USS and μCT.

	USS		μCT	
	Suprarenal Aorta	Infrarenal Aorta	Suprarenal Aorta	Infrarenal Aorta
Aortic volume	7.57	3.254	8.02	3.8
	7.52	4.558	8.49	4.47
	7.28	2.707	8.84	3.75
	6.308	2.879	6.58	3.37
	8.79	3.634	8.49	5.03
Mean ± SEM	7.5 ± 0.39	3.4 ± 0.32	8.1 ± 0.39	4.1 ± 0.29

**Table 2:** Reproducibility and Consistency of Aortic Region Measurements Using μCT and USS.

Aortic Region	Measurement Modality	Mean APmax (mm)	Standard Deviation (mm)	Correlation (R^2^)	*p*-Value
Suprarenal Aorta	μCT—Inter Observer	1.18	0.10	0.84	0.02
	μCT—Intra Observer	1.18	0.10	0.97	0.001
	USS—Intra Observer	1.03	0.02	0.86	0.02
Infrarenal Aorta	μCT—Inter Observer	0.83	0.02	0.90	0.01
	μCT—Intra Observer	0.83	0.02	0.99	0.0002
	USS—Intra Observer	0.67	0.02	0.82	0.03

## Data Availability

The raw data supporting the conclusions of this article will be made available by the authors on request.
